# Pollution induces epigenetic effects that are stably transmitted across multiple generations

**DOI:** 10.1002/evl3.273

**Published:** 2022-02-03

**Authors:** Ewan Harney, Steve Paterson, Hélène Collin, Brian H.K. Chan, Daimark Bennett, Stewart J. Plaistow

**Affiliations:** ^1^ Evolution, Ecology and Behaviour, Institute of Infection, Veterinary and Ecological Sciences University of Liverpool Liverpool L69 7ZB United Kingdom; ^2^ Current address: Institute of Evolutionary Biology (CSIC‐UPF) CMIMA Building Barcelona 08003 Spain; ^3^ Current address: Faculty of Biology, Medicine and Health The University of Manchester Manchester M13 9PT United Kingdom; ^4^ Molecular and Physiology Cell Signalling, Institute of Systems, Molecular and Integrative Biology University of Liverpool Liverpool L69 7ZB United Kingdom

**Keywords:** Cytosine methylation, *Daphnia pulex*, ecotoxicology, gene body methylation, transgenerational epigenetic inheritance

## Abstract

It has been hypothesized that the effects of pollutants on phenotypes can be passed to subsequent generations through epigenetic inheritance, affecting populations long after the removal of a pollutant. But there is still little evidence that pollutants can induce persistent epigenetic effects in animals. Here, we show that low doses of commonly used pollutants induce genome‐wide differences in cytosine methylation in the freshwater crustacean *Daphnia pulex*. Uniclonal populations were either continually exposed to pollutants or switched to clean water, and methylation was compared to control populations that did not experience pollutant exposure. Although some direct changes to methylation were only present in the continually exposed populations, others were present in both the continually exposed and switched to clean water treatments, suggesting that these modifications had persisted for 7 months (>15 generations). We also identified modifications that were only present in the populations that had switched to clean water, indicating a long‐term legacy of pollutant exposure distinct from the persistent effects. Pollutant‐induced differential methylation tended to occur at sites that were highly methylated in controls. Modifications that were observed in both continually and switched treatments were highly methylated in controls and showed reduced methylation in the treatments. On the other hand, modifications found just in the switched treatment tended to have lower levels of methylation in the controls and showed increase methylation in the switched treatment. In a second experiment, we confirmed that sublethal doses of the same pollutants generate effects on life histories for at least three generations following the removal of the pollutant. Our results demonstrate that even low doses of pollutants can induce transgenerational epigenetic effects that are stably transmitted over many generations. Persistent effects are likely to influence phenotypic development, which could contribute to the rapid adaptation, or extinction, of populations confronted by anthropogenic stressors.

**Impact Summary**

The epigenome—the collection of proteins and chemicals that provide structure to the DNA in the genome and regulate its expression—is sensitive to environmental and anthropogenic stresses. These changes can have important effects on the phenotypes of organisms, and there is increasing evidence that stress‐induced changes to the epigenome are transmitted across generations. However, few studies have demonstrated that these effects are transmitted more than one or two generations, limiting their potential to influence evolution. One important epigenetic mark is the methylation of cytosine in DNA. Here, we looked for signs of persistent differential methylation following pollutant exposure in the water flea *Daphnia pulex*.

*Daphnia* is well suited to these types of studies because it often reproduces asexually. It is therefore possible to compare many genetically identical individuals across different pollutants. Here, we exposed replicated populations to three different freshwater pollutants for 7 months (>15 generations), and then switched half of the populations to clean water for 7 months (>15 generations). At the end of the experiment, we compared DNA methylation of populations that had experienced continual pollutant exposure, populations that had experienced the pollutant followed by clean water, and control populations that had never experienced the pollutant.

We found that all three pollutants led to changes to DNA methylation. Importantly, some of these changes were detectable in both the continually treated and the switched to water treatments, implying that these persistent modifications were stably passed down through the generations, even in the absence of the pollutant. A follow‐up experiment confirmed that three generations after pollutant exposure, phenotypic effects were still detectable. The presence of both stable epigenetic transmission over many generations and phenotypic consequences suggests these effects have the potential to influence evolutionary processes either by contributing a second, environment‐induced source of heritable phenotypic variation or by altering the epigenome in a way that alters the probability that an environmentally induced trait becomes genetically fixed.

Agricultural, industrial, and household activities release thousands of chemical compounds into soil or drainage systems, which then contaminate freshwater streams, rivers, and lakes. These compounds are potentially toxic, affecting the health of individuals, the dynamics of populations, and the productivity of ecosystems (Walker et al. 2012). Current risk assessment frameworks focus on understanding the impact that acute and chronic exposure to pollutants has on the biology of directly exposed individuals (Walker et al. 2012). However, it is hypothesized that epigenetic inheritance mechanisms such as histone modification, the production of noncoding RNAs, and DNA methylation can transmit the effects of pollutant exposure to future nonexposed generations, potentially generating long‐term effects (Anway et al. 2005; Baccarelli and Bollati 2009; Vandegehuchte and Janssen 2011; Mirbahai and Chipman 2014). The ability to pass environment‐induced information from one generation to the next via epigenetic inheritance is well‐established for plants (Cubas et al. 1999; Johannes et al. 2009; Verhoeven et al. 2010; Quadrana and Colot 2016) but its generality is more contentious in animals (Anway et al. 2005; Jablonka and Raz 2009), where the clearer segregation of germline and soma (Jablonka 2012; Lanfear 2018) may limit the opportunity for environmentally induced epigenetic changes to be transmitted. The roles and relative importance of different epigenetic mechanisms also vary significantly between taxa (Head 2014; Lewis et al. 2020), and their transmission depends on factors such as the mode of reproduction (Richards 2006) and the way that primordial germ cells (PGCs) are derived (Navarro‐Martín et al. 2020). Nonetheless, some epigenetic changes such as methylation persist after global epigenome reprogramming associated with PGC development (Kremsky and Corces 2020), and there appears to be a critical window during early development during which environmental perturbations may result in changes to the germline epigenome (Sheldon et al. 2020).

What is less well understood is if modifications to the epigenome of the germline are transmitted to subsequent generations. In studies of organisms with internal fertilization (i.e., viviparous and true oviparous species, but not externally fertilizing ovuliparous species), it is not only the developing offspring but potentially their germline (nascent grand‐offspring) that can be directly exposed to environmental changes experienced by fertilized or gravid mothers. To demonstrate true transgenerational epigenetic inheritance in these cases, environmentally induced changes should ideally be observed beyond the grand‐offspring generation (Bossdorf et al. 2008; Heard and Martienssen 2014). Therefore, only by studying epigenetic dynamics over more than three generations can we quantify the possible impact of environmental and anthropogenic stressors on the evolution of populations (Burggren 2015). Despite the challenges associated with long‐term epigenetic studies, there is now mounting evidence that epigenetic modifications such as DNA methylation can be inherited beyond those generations with a direct experience of the environmental signal (Hanson and Skinner 2016; Demoinet et al. 2017; Klosin et al. 2017; Kamstra et al. 2018; Beck et al. 2021).

Although our understanding of how persistent epigenetic effects are translated into measurable consequences for phenotypic development has improved recently (Demoinet et al. 2017; Kamstra et al. 2018), often the functional importance of these modifications is unclear (e.g., Beck et al. 2021). Characterizing the genomic context of epigenetic changes (are changes associated with promoters/genes, and if so which genes?; Major et al. 2020) and the direction of changes (does methylation increase or decrease?; Metzger and Schulte 2017) remain important steps in determining how the epigenome might be influencing phenotypic development. Furthermore, identifying whether there are phenotypic consequences to these changes is essential in understanding how environmentally induced persistent modifications will affect the evolution of populations (Adrian‐Kalchhauser et al. 2020).

Here, we use the freshwater cladoceran *Daphnia pulex* to test whether three common environmental pollutants can induce persistent epigenetic effects lasting many generations. *Daphnia* species have long been used as sentinel species to indicate water quality and ecosystem health in freshwater systems (Shaw et al. 2008). But they are now also increasingly used as epigenetic model systems because epigenetic effects can easily be disentangled from genetic effects (Ebert 2011; Harris et al. 2012). This derives from the fact that *Daphnia* species normally reproduce via parthenogenesis, resulting in clonal lineages that allow phenotypic comparison of genetically identical individuals across a range of different environments. Several previous studies have exploited this aspect of *Daphnia* biology and demonstrated that biotic (Asselman et al. 2015) and abiotic (Vandegehuchte et al. 2009, 2010) stressors alter global patterns of DNA methylation. However, many of these studies did not last long enough to quantify the effects of exposure for future nonexposed generations (Vandegehuchte and Janssen 2011; Burggren 2015; Shaw et al. 2017). Jeremias et al (2018) did find transmission of environmentally induced differential methylation (DM) to the first nondirectly affected generation (the great grand offspring), but it is still unclear whether such modifications might be stable in the long term. Furthermore, although DM of specific genes has been observed and provides some ideas about the genomic functions affected by environmentally induced methylation in *Daphnia* (Asselman et al. 2017), less is known about broad‐scale changes in groups of genes, nor whether pollutant‐induced modifications leads to increases or decreases in methylation.

Using whole genome bisulfite sequencing, we examine the genome‐wide effects of chronic pollutant exposure on the induction and stability of cytosine methylation patterns within the *D. pulex* genome over many generations. We explore the functional importance of epigenetic modifications through analysis of their genomic context, whether the percentage of methylated reads increased or decreased, and gene function analysis. The pollutants chosen were cadmium, as a representative of heavy metal pollution; glyphosate, as a representative of herbicide pollution; and 4‐nonylphenol, which is an endocrine disruptor commonly found in detergents. We also quantify the effects of these pollutants on individual development, growth, and life‐history traits in the descendants of exposed animals.

## Materials and Methods

### GROWTH OF DAPHNIA FOR METHYLATION ANALYSIS

**Figure 1:**
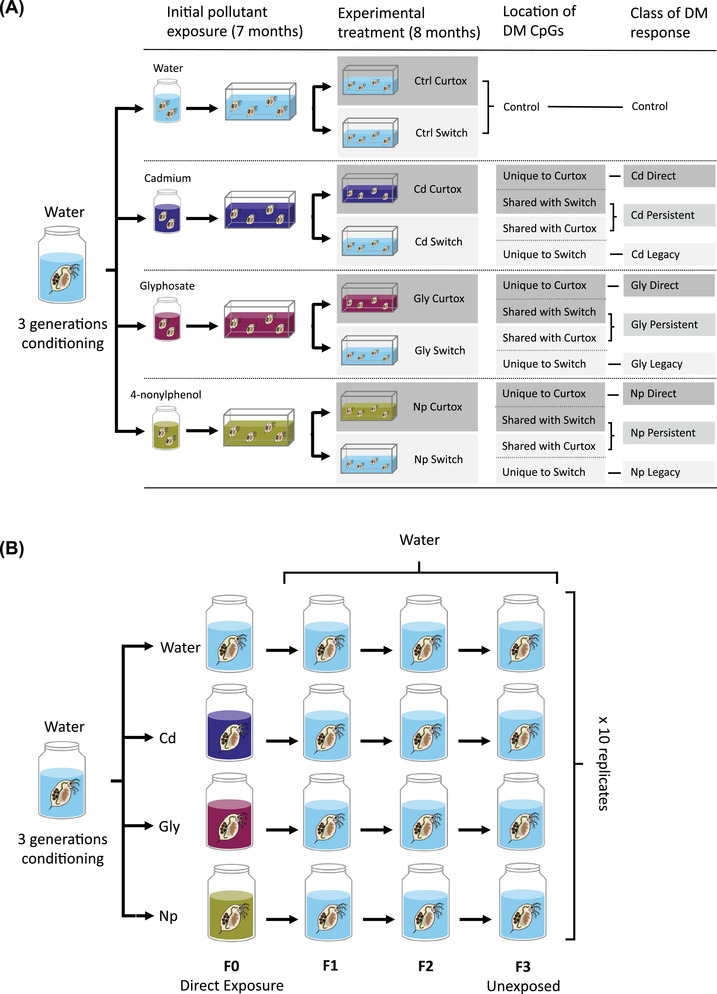
(A) Conditioned *D. pulex* were maintained in one of three pollutants or a clean water control for 7 months before populations were split and either subjected to a continued pollutant treatment (*curtox*—5 replicates) or clean water (*switch*—5 replicates) for a further 7 months. Differential methylation (DM) analysis compared methylation of CpGs in the curtox and switch treatments (three replicates of each) to the combined controls (six replicates). Significantly DM CpGs (FDR < 0.05) were then grouped according to whether they were found only in the curtox treatment (*direct* effects), found in both treatments (*persistent* effects), or found only in the switch treatment (*legacy* effects). Here and throughout, water control, Cd, Gly, and Np are designated by pale blue, purple‐blue, magenta‐red, and gold‐yellow, respectively. (B) To assess whether pollutants were influence phenotypes in a completely unexposed generation, conditioned *D. pulex* were transferred to one of three pollutants or a control for a single generation before being returned to clean water for a further three generations.

*Daphnia pulex* clone LL14 was isolated from a population on Anglesey, United Kingdom (53°14ʹ45ʺN, 4°08ʹ12ʺW) and maintained as a stock culture for over 6 months in a controlled temperature incubator at 21 ± 1°C on a 14:10 L:D cycle. Under the same temperature and lighting regime, 500‐mL jars were set up containing hard water ASTM enriched with an organic extract (Marinure) (Baird et al. 1989) plus 2 mL of either a water control or one of three sterile stock solutions of a pollutant giving the following final concentrations: 0.05 μg L^–1^ CdCl_2_, 100 μg L^–1^ glyphosate, or 10 μg L^–1^ 4‐nonylphenol (one jar per treatment). The mild doses of pollutant fall within ranges observed in natural European freshwater systems (0.001–0.1 μg L^–1^ cadmium: Pan et al. 2010; 0.2–650 μg L^–1^ glyphosate: Székács and Darvas 2018; 0.14–795 μg L^–1^ 4‐nonylphenol: Hong et al. 2020) and were established in a preliminary study of multiple clones (including LL14) as the minimum dose tested at which *D. pulex* still produced enough offspring to setup future generations but otherwise had a demonstrable effect on offspring life histories (Fig. S1; Table S1). The jars were fed medium food (120,000 cells mL^–1^ day^–1^ of semicontinuous cultured *Chlorella vulgaris*) three times per week and animals were placed in new jars every week. Every other week, the population in each jar was thinned by 50% to maintain the populations below carrying capacity. After 4 months, the *Daphnia* in each jar were transferred into 10‐L tanks, filled to a level of 5 L, to allow for large numbers of individuals to be produced (one tank per treatment). The food regime was reduced to low food (40,000 cells mL^–1^ day^–1^ of *C. vulgaris*) to prevent populations overshooting their carrying capacity and initiating sexual reproduction. Populations were re‐polluted each week and put into new tanks every other week. After 3 months, each treatment tank was used to set up five replicate tanks that were put back in the same pollutant (current toxin or *curtox* treatment), and five replicate tanks that were put back in control tanks with no pollutant (switched‐to‐control or *switch* treatment). After 7 months under this regime, all 40 tanks were harvested and DNA was extracted (Fig. 1A).

### MOLECULAR METHODS

A volume of approximately 750 μL of *Daphnia* were collected by filtration, then picked with a pipette into a 1.5‐mL tube and flash frozen in liquid nitrogen. Samples were ground with a plastic pestle in 500 μL of lysis buffer (50 mM TrisHCl, 25 mM NaCl, 25 mM EDTA at pH 8.0, 0.1% SDS, 0.1 M DTT all from Sigma Aldrich, St. Louis, MO [U.S.A.]) plus 1 μL of Proteinase K (18 mg mL^–1^, Sigma Aldrich), gently vortexed and incubated for 90 minutes at 55°C. DNA was then extracted twice with 500 μL of chloroform isoamyl alcohol solution (Sigma Aldrich, St. Louis, MO [U.S.A.]) followed by centrifugation at 14,000 rpm and precipitated at −20°C by adding 850 μL of 100% molecular grade ethanol (Sigma Aldrich, St. Louis, MO [U.S.A.]) and 30 μL of 3M NaOAc at pH 5.2 (Sigma Aldrich, St. Louis, MO [U.S.A.]) followed by centrifugation at 14,000 rpm. DNA was resuspended in 30 μL of water plus 1 μL of RNAse A (Sigma Aldrich, St. Louis, MO [U.S.A.]) and incubated for 30 minutes at room temperature. DNA was then purified using an Agencourt^®^ Ampure^®^ XP beads purification kit following manufacturer's instructions (Beckman Coulter, Brea, CA [U.S.A.]). Quality and quantity of DNA were assessed using a Qubit fluorometer (Invitrogen, Waltham, MA [U.S.A.]), DNA Nanodrop (ThermoFisher, Waltham, MA [U.S.A.]), and Bioanalzer (Agilent, Santa Clara, CA [U.S.A.]). For each treatment combination, three DNA samples passing quality control were used to create bisulfite‐treated libraries using Epicenter Methyl‐Seq kits (Epicentre Biotechnologies, Madison, WI [U.S.A.]) according to manufacturer's instructions (24 samples total). Libraries were sequenced as 100‐bp paired‐end reads on an Illumina HiSeq2000 platform with approximately 44 million reads per sample.

### CHARACTERISATION OF THE METHYLOME

A reference genome specific to clone LL14 was created from genomic DNA isolated using Qiagen Genomic Tip (Qiagen, Hilden [Germany]) according to manufacturer's instructions and an Illumina TruSeq library (Illumina, San Diego, CA [U.S.A.]) with 500‐bp insert sequenced with 250‐bp paired‐end reads on an Illumina MiSeq platform. Reads were assembled with Newbler assembler version 2.6 and contigs were placed on the *D. pulex* genome assembly (GCA_000187875.1) and annotated using MAKER (Campbell et al. 2014), with orthology of genes determined using reciprocal Blast within InParanoid version 4 (O'Brien et al. 2005).

Bisulfite converted reads were then mapped to the LL14 reference using Bismark, duplicate reads were removed, and counts of methylated and unmethylated cytosines extracted. Only CpG sites with ≥10 reads (methylated + unmethylated) per sample were considered. Genome‐wide methylation was calculated using the six control samples, and overrepresentation of heavily methylated CpGs (in which >50% of reads from all controls were methylated) was calculated for exons, introns, and 5ʹ promoters (here defined as 2 kb upstream of the first exon) using *t*‐tests. We explored the genomic context of methylation by plotting the density of CpGs 1 kb either side of heavily methylated (>50%) CpGs. For CpGs with variable methylation (1% < methylated reads < 99%, calculated across all controls), repeatability of methylation (the ratio of methylated to unmethylated reads) in a random sample of 10,000 CpG sites was calculated and compared to a null distribution using the *rptR* package (Stoffel et al. 2017) in *R* (verion 3.6.0; R Development Core Team 2019). An average methylation landscape for genomic features (exons, introns, 5ʹ promoters, and 3ʹ regions 2 kb downstream of the last exon) was calculated using the method of Kvist et al. (2018).

### DM ANALYSIS OF CpGs

DM analysis was carried out separately for each pollutant. Prior to running the analyses, two additional filters were applied: (i) CpGs with excessive missing values were removed (>2 among the six control samples, and/or >1 in the three curtox and/or >1 in three switch treatment samples); (ii) CpGs with very low (<1%) or very high (>99%) methylation were removed (percentage methylation was calculated across all control, curtox, and switch samples). CpGs that passed these filters were analyzed using generalized linear models with a binomial distribution in *R* to test for the effect of treatment on methylation counts. *P*‐values were corrected using a false discovery rate (FDR), with FDR < 0.05 deemed significant. For each pollutant, DM CpGs were split into those with increased methylation and those with decreased methylation, and the mean and standard deviation change in methylation were calculated (i.e., the percentage of methylated reads in the treatment minus the percentage of methylated reads in the control). To visualize differences among treatments and pollutants, multidimensional scaling of DM CpG sites was carried out using the *cmdscale* function.

To identify similarities and differences in methylation between the curtox and switch treatments, DM CpGs were classed as having one of three response types (Fig. 1A), depending on whether they were found (a) only in the curtox treatment (“direct” responses); (b) only in the switch treatment (“legacy” responses); or (c) in both treatments (“persistent” responses). Fisher's exact tests were performed to determine whether DM CpGs associated with direct, legacy, or persistent responses were overrepresented in genomic features (exons, introns, or 5ʹ promoter regions). Focusing on the DM CpGs found in these genomic features (as well as 3ʹ downstream regions), we assessed (a) whether increases and decreases in methylation were consistent among response types, and (b) whether DM CpG methylation percentage in untreated controls differed among response types. A linear model was used to test for the significance of response type and gene feature (plus their interaction) on change in methylation. The emmeans package (Lenth 2020) was used to determine whether change in methylation was significantly lower or higher than zero (indicating a consistent trend toward decreased or increased methylation). A generalized linear model with a quasibinomial distribution (logit link function) was used to test for significance of response type and gene feature on methylation level of DM CpGs in controls.

### METHYLATION OF GENES

We continued to use the three classes of response (direct, legacy, and persistent) in the analysis of genes associated with DM CpGs, which we term DM genes. Genes that contained exclusively curtox DM CpGs were “direct” DM genes, those that contained exclusively switch DM CpGs were “legacy” DM genes, and genes containing both curtox and switch DM CpGs (even if the CpGs themselves were distinct) were termed “persistent” DM genes. For each pollutant, the number of DM genes in each response type were visualized with scaled Venn diagrams, as was the overlap between pollutants for direct, persistent, and legacy DM genes.

Finally, for each pollutant, putative functions of annotated direct, legacy, and persistent DM genes were assessed with the Panther Overrepresentation Test, using the panther GO Biological Process complete database (Mi et al. 2013) and a *Daphnia pulex* reference list containing all uniprot IDs associated with CpGs for which there was sufficient coverage (≥10 bisulfite reads). Redundant GO terms were removed using Revigo (Supek et al. 2011) with allowed similarity set to 0.5, and log‐fold change GO term overrepresentation was visualized in *R*, with the most generic GO terms removed (those associated with >2000 *D. pulex* genes in the reference list). To examine whether there were broad differences between functions of genes with increased or decreased methylation, we carried out a further functional enrichment comparing DM genes with increased and decreased methylation. Carrying out the analysis for each pollutant separately and considering direct, persistent, and legacy responses resulted in very few significantly overrepresented GO terms (zero for most pollutant/response combinations), so lists of DM genes were aggregated, such that the difference between curtox and switched treatments (with all pollutants combined) was considered.

### INDIVIDUAL LIFE‐HISTORY ANALYSIS OF DAPHNIA

In a separate follow‐up experiment, we tested whether low doses of the pollutants had persistent effects on the growth, development, and life histories of descendant *Daphnia* (three generations after exposure). Prior to experimentation, clone LL14 was conditioned in individual jars for three generations to control for maternal effects (see Plaistow and Collin 2014 for methods) and offspring from the third clutch of third‐generation mothers were used to set up the experiment. Ten replicate animals in each treatment were kept individually in a 175‐mL jar containing 150 mL of hard water ASTM enriched with an organic extract (Marinure) (Baird et al. 1989). Each individual was fed 200,000 cells mL^–1^ day^–1^ of *C. vulgaris* (high food) when the media in each jar was changed. Parental lines were exposed to 2 mL of either a water control or one of three sterile stock solutions of a pollutants at the following final concentrations: CdCl_2_ (0.01 μg L^–1^), glyphosate (50 μg L^–1^), or 4‐nonylphenol (25 μg L^–1^). Following exposure to pollutant in the parental generation, all subsequent offspring were reared in a common garden environment (with no pollutant) and assayed for a further three generations (Fig. 1B). Ten replicates of third‐clutch offspring were used to set up each new generation and were maintained and treated individually using the same protocol described above for control lines.

All individuals in each generation were photographed as neonates and then every time they molted using digital cameras (Canon EOS 600D and 1100D) connected to Leica dissecting microscopes (MZ6 and M60). Body size was measured from the ventral base of the tail spine to the anterior edge of the carapace using the ImageJ 1.46q image analysis package (Rasband 1997). Developmental stage was recorded, with individuals considered as mature once eggs were observed in the brood pouch. For each clutch that females produced (up to the third clutch), the number of offspring was counted. Consequently, each individual in the experiment had five life‐history traits measured: size at maturity (mm), age at maturity (days), size upon dropping the third clutch (mm), age upon dropping the third clutch (days), and total fecundity (total offspring produced from three clutches). For both generations studied (F0 and F3), the effect of pollutant treatment was analyzed independently using analysis of variance (ANOVA). Because this involved carrying out 10 individual ANOVAs, FDR was applied to correct for multiple testing. For traits in which treatment was found to be significant at our statistical cutoff (FDR < 0.05), Dunnett's Multiple Comparison tests were used to determine which treatments differed significantly from controls. Statistical analyses were performed in *R* and Dunnett's tests were carried out using the *DescTools* package (Signorell et mult. al 2021).

## Results

### WHOLE GENOME BISULFITE SEQUENCING

**Figure 2:**
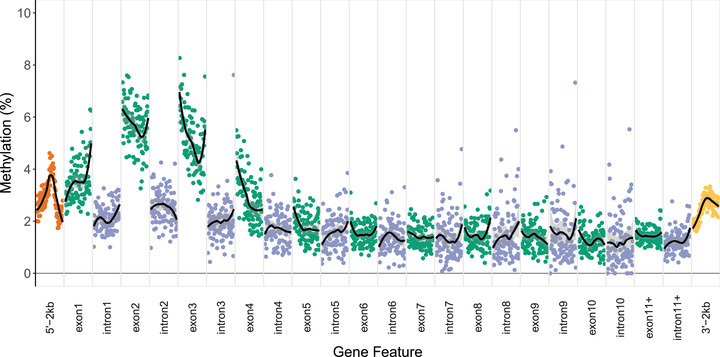
Average methylation landscape across genomic features of 13,473 *D. pulex* genes in a single control sample. Genomic features include exons 1–10 and 11+, introns 1–10 and 11+, 2 kbp upstream of the first exon, and 2 kb downstream of the last exon. Individual points represent the average methylation of CpGs with similar locations relative to the start of genomic features. Loess fits provide an overall picture of average methylation within different features.

An average of 44.5 million paired‐end reads (± 3.6 million; 95% confidence interval [CI]) were sequenced per sample: 44.46% (± 3.33%; 95% CI) of these reads were successfully mapped to the LL14 genome assembly, providing an average genome‐wide coverage of 26.5× (± 2.97; 95% CI) after deduplication. Whole genome bisulfite sequencing of control *D. pulex* revealed low genome‐wide methylation (1.87% ± 0.05%; 95% CI) with just 0.71% (± 0.04%, 95% CI) of CpG sites heavily (>50%) methylated. Heavily methylated sites were overrepresented in exons or putative promoter regions (Table 1) and tended to be in regions with relatively low CpG density (Fig. S2). Across the genome, methylation was generally higher in earlier exons (exons 1–4) than later exons or introns (Fig. 2). Repeatability of methylation in control samples was somewhat low for CpGs with 1% < methylation < 99% (*R* = 0.069), although it was significantly greater than predicted by a null distribution (*P* = 0.002; Fig. S3). Repeatability increased when using CpGs with 5% < methylation < 95%, and 10% < methylation < 90% (*R* = 0.118, *P* = 0.002 and *R* = 0.154, *P* = 0.002, respectively; Fig. S3); however, we chose to use CpGs with 1% < methylation < 99% as our filter for DM analysis, due to the low average methylation of genomic features (Fig. 2).

**Table 1:** Methylation of CpG sites by gene feature (exon, intron, promoter) across control samples. Sites with at least 50% methylation were significantly overrepresented within exons and promoters. Promoters here are defined as sites within 2 kb upstream of the transcriptional start position

	All CpG Sites	>50% Methylation		Odds Ratio	*P*‐value
Genome	5,449,861	39,099	(0.7%)	–	
Exon	1,197,309	20,380	(1.7%)	2.40	0.002
Intron	701,919	5442	(0.8%)	1.09	0.660
Promoter	766,477	14,361	(1.9%)	2.64	0.001

### DM OF CpGs

DM analysis of 684,619–873,241 CpGs that passed our two filters (filters were applied separately for each pollutant) found a total of 6508 CpG sites to be differentially methylated in at least one treatment, with considerable overlap between treatments and pollutants. We found 1469 (Cd), 2423 (Gly), and 1689 (Np) CpG sites were differentially methylated (FDR < 0.05) in curtox samples relative to controls, and 1408 (Cd), 1373 (Gly), and 1475 (Np) CpGs sites were differentially methylated (FDR < 0.05) in switch samples relative to controls. DM CpGs showed on average 17.3–31.8% difference in methylation relative to controls (Table 2; Fig. S4), depending on the pollutant, treatment, and whether methylation was increasing or decreasing. Decreases in methylation (24.9–31.8%) tended to be larger than increases in methylation (17.3–25.5%).

**Table 2:** Mean and standard deviation change in methylation percentage of significantly differentially methylated CpGs for different pollutant/treatment combinations (relative to control). The change in methylation is the percentage of methylated reads in the treatment reads minus the percentage of methylated reads in the control. Decreases in methylation tended to be larger than increases in methylation

Pollutant	Treatment	Direction of Change (Relative to Control)	Number of CpGs	Mean Change	SD
Cd	Cd curtox	+	707	21.1	10.1
		−	762	–29.0	13.0
	Cd switch	+	744	25.5	11.2
		−	664	–28.9	14.0
Gly	Gly curtox	+	2034	17.3	7.6
		−	389	–30.1	12.3
	Gly switch	+	792	18.6	8.6
		−	581	–24.9	11.7
Np	Np curtox	+	1122	22.2	13.3
		−	567	–26.8	12.4
	Np switch	+	1046	22.9	11.9
		−	429	–31.8	12.8

**Figure 3:**
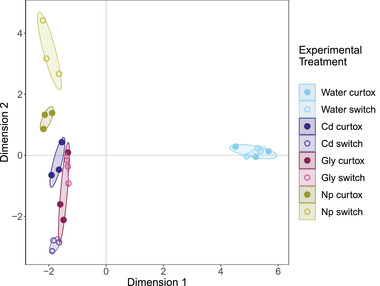
Multidimensional scaling plot of 2543 DM CpGs sites. Confidence ellipses (95%) are drawn around treatment groups. All pollutant treatments separate from water controls along the first dimension, whereas differences between pollutants (particularly Cd and Np in the switch treatment) separate from one another along the second dimension.

Multidimensional scaling of 2543 differentially methylated CpG sites (for which there were no missing values) revealed consistent patterns of methylation between the two different controls (Fig. 3), justifying our decision to group them together. Methylation patterns were also relatively consistent within pollutant treatments. All curtox and switch samples were clearly separated from control samples along the first dimension, whereas the second dimension highlighted differences between pollutants as well as differences between the curtox and switch treatments for Cd and Np.

**Figure 4:**
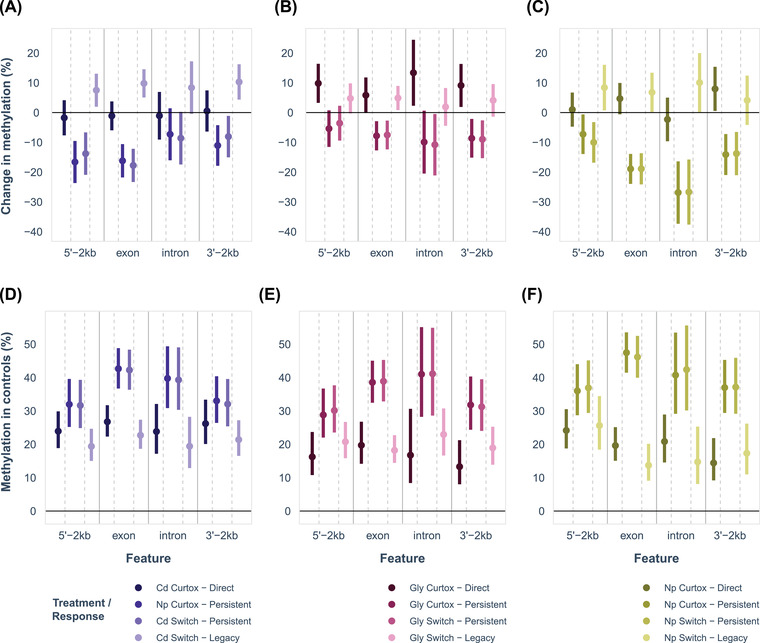
(A–C) Avergae increases and decreases in methylation (percentage of methylated reads in treatment minus percentage of methylated reads in control) with 95% confidence intervals following pollutant exposure for DM CpGs across exons, introns, 5ʹ—2kb (2 kbp upstream of first exon), and 3ʹ—2 kb (2 kb downstream of last exon) for (A) cadmium, (B) glyphosate, and (C) 4‐nonylphenol. DM CpGs are classified according to their treatment (curtox or switch) and whether they were unique to their treatment (curtox—direct, switch—legacy) or found in both treatments (curtox persistent, switch persistent). (D–F) Average methylation (%) with 95% confidence intervals of these CpGs in control samples.

Because many DM CpGs were shared between the curtox and switch treatments, we subdivided CPGs into three classes: direct DM CpGs (DM in curtox only), persistent DM CpGs (DM in both treatments), and legacy DM CpGs (DM in switch only). For all three pollutants, persistent DM CpGs were overrepresented in promoters and exons (Table 3). Legacy DM CpGs were also overrepresented in promoters and exons in most cases, whereas results were less consistent among direct DM CpGs. For all pollutants, persistent DM CpGs showed consistent decreases in methylation, whereas legacy DM CpGs showed consistent increases in methylation (Fig. 4A–C; Tables S2 and S3). For Gly, direct CpGs also showed consistent increases in methylation. Effects did not differ among genomic features in Cd or Gly, but exons and introns were less methylated than 5ʹ and 3ʹ regions in Np (Table S3). Patterns of decreasing and increasing methylation seem to reflect the baseline methylation in control samples (Fig. 4D–F). For all pollutants, persistent DM CpGs had greater baseline methylation in the controls than direct and legacy DM CpGs (Tables S2 and S4). Among control samples, genomic feature was only a significant factor for Cd, for which methylation of exons was higher than that of 5ʹ promoters (Table S4).

**Table 3:** CpG sites exhibiting significant (FDR < 0.05) differential methylation relative to water controls. DM was initially calculated for the current toxin (curtox) and switched to water (switch) treatments, and then DM CpGs (FDR < 0.05) were grouped according to whether they were found only in the curtox treatment (direct effects), found in both treatments (persistent effects), or found only in the switch treatment (legacy effects). Only sites with a mean methylation level between 1% and 99% were tested. Number of sites exhibiting significant differential methylation are shown for the whole genome and within three genomic features (promoters, exons, and introns). Numbers in brackets are expected numbers for genomic features based on their proportion of the genome. For genes located close to one another, some sites may be counted in both promoter and exon or intron categories where they overlap. Observed values in bold designate significant overrepresentation, and values in italics designate significant underrepresentation. Significance level is indicated by asterisks: *FDR < 0.05; **FDR < 0.01; ***FDR < 0.001

			Promoters			Exons			Introns		
Pollutant	Contrast	Genome	Obs.	(exp.)		Obs.	(exp.)		Obs.	(exp.)	
Cd	Total CpGs tested	873,241	145,393			247,796			124,739		
	DM: Direct	931	**322**	(155)	***	**393**	(264)	***	141	(133)	
	DM: Persistent	538	**217**	(90)	***	**291**	(153)	***	**107**	(77)	**
	DM: Legacy	870	**299**	(145)	***	**437**	(247)	***	103	(124)	
Gly	Total CpGs tested	791,115	122,188			210,156			106,022		
	DM: Direct	1951	*157*	(301)	***	*252*	(518)	***	*84*	(261)	***
	DM: Persistent	472	**192**	(73)	***	**262**	(125)	***	75	(63)	
	DM: Legacy	901	**283**	(139)	***	**379**	(239)	***	140	(121)	
Np	Total CpGs tested	684,619	109,714			184,515			95,403		
	DM: Direct	949	**222**	(152)	***	272	(256)		*106*	(132)	*
	DM: Persistent	740	**184**	(119)	***	**238**	(199)	*	87	(103)	
	DM: Legacy	735	**161**	(118)	***	214	(198)		89	(102)	

### PATTERNS OF METHYLATION AMONG GENES

**Figure 5:**
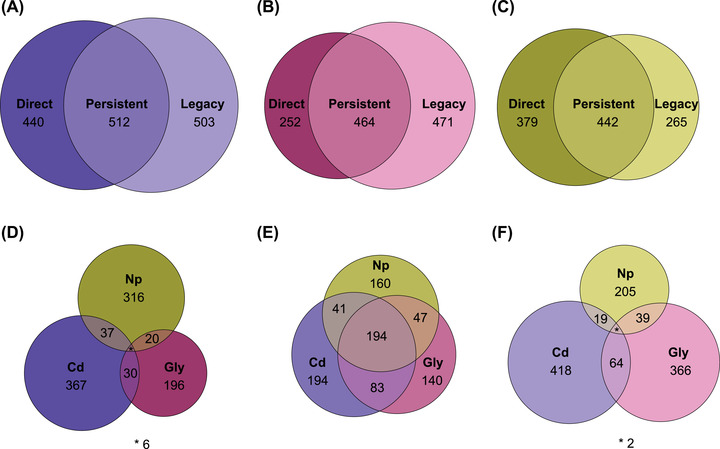
Scaled Venn diagrams showing overlap in DM genes between treatments (A–C) and pollutants (D–F). Overlaps between DM genes in the current toxin treatment (left‐hand side) and switched treatment (right‐hand side) are shown for (A) cadmium, (B) glyphosate, and (C) 4‐nonylphenol pollutants. Sets of DM genes are termed “Direct” (current toxin only), “Persistent” (found in both treatments), and “Legacy” (switched treatment only). Subsequently, (D) Direct, (E) Persistent, and (F) Legacy sets were then used to identify overlap in DM genes between the three pollutants.

Aggregating direct, persistent, and legacy DM CpGs into direct, persistent, and legacy DM genes revealed that for Cd and Np, more genes were classed as persistent than direct or legacy (Fig. 5A, C), although for Gly legacy genes were more numerous (Fig. 5B). Persistent DM genes showed much greater levels of overlap between pollutants than direct or legacy DM genes (Fig. 5D–F), suggesting that genes that were differentially methylated in both curtox and switched treatments may be reflective of general pollutant stress responses, whereas the direct and legacy responses may be more pollutant specific.

**Figure 6:**
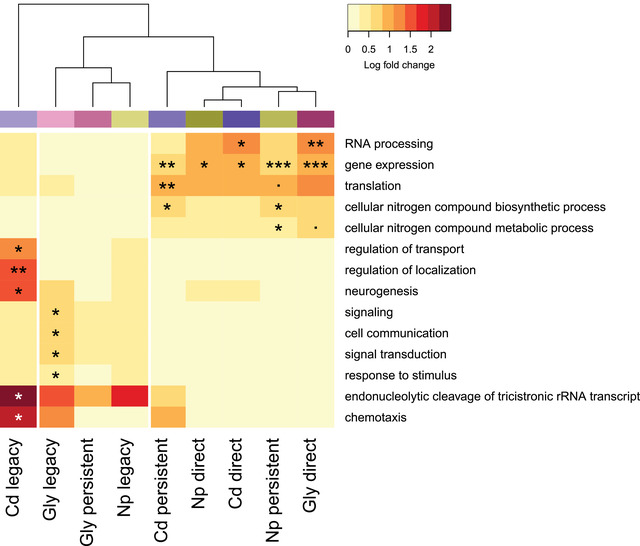
Overrepresentation of gene ontology (GO) biological process terms in Direct, Persistent, and Legacy sets of DM genes for all pollutants. Highly generic terms (GO terms with associated with ≥2000 *D. pulex* genes) were removed. Underrepresented terms (fold change in term enrichment <1, none of which were significant) were set to 1 to better visualize changes in significantly overrepresented terms. Color intensity denotes log‐fold change in term enrichment, which was used to cluster terms and pollutant/response classes (also indicated by the colored bar underneath the dendrogram). Significance level is indicated by asterisks: **·** FDR < 0.1; * FDR < 0.05; ** FDR < 0.001; *** FDR < 0.0001.

Using a background list of 11,007 uniprot IDs for which there was sufficient bisulfite read coverage, overrepresentation analysis of nongeneric GO biological processes revealed only weak patterns of functional enrichment for most response types. The most striking overrepresentation was for GO terms associated with the Cd legacy response (Fig. 6), in which endonucleolytic cleavage of tricistronic rRNA transcripts (SSU‐rRNA, 5.8S rRNA, LSU‐rRNA) (GO:0000479) and chemotaxis (GO:0006935) were very highly overrepresented (log fold change [LFC] > 1.9). Both these terms were also rather specific (comprising <50 uniprot IDs in the background list). Furthermore, the GO terms neurogenesis (GO:0022008) and regulation of localization (GO:0032879) were also strongly overrepresented for Cd legacy responses (LFC > 1.4, comprising <150 uniprot IDs in the background list). For most other pollutant/responses combinations, significant overrepresentation tended to be weaker (LFC values of 0.5–1.2) and involve less specific GO terms (>200 uniprot IDs in the background list). The analysis also clustered the direct responses from all three pollutants, together with persistent Cd and Np responses. The GO term gene expression (GO:0010467) was significantly overrepresented in all five of these responses, and the GO terms RNA processing (GO:0006396) and translation (GO:0006412) were also significantly overrepresented in one or two of these responses and appeared to be somewhat overrepresented in the others (Fig. 6), although not to a significant degree. Gly and Np legacy responses together with the Gly persistent response also formed a cluster, although among these responses only the Gly legacy response was associated with any significantly overrepresented GO terms, and these showed weak log‐fold increases in overrepresentation.

Similarly, functional analysis of genes showing increases in methylation with those showing decreases in methylation in the curtox and switch treatments did not reveal strong patterns of overrepresentation (Fig. S5), with only six nongeneric GO terms showing significant overrepresentation. However, the analysis did cluster methylation responses by direction (increased or decreased methylation). Furthermore, among increased methylation responses, the switched treatment showed stronger overrepresentation than the curtox treatment, notable in the GO term posttranscriptional regulation of gene expression (GO:0010608), whereas among decreased methylation responses, overrepresentation was stronger for curtox treatments than switched treatments.

### PHENOTYPIC CONSEQUENCES OF ANCESTRAL POLLUTION EXPOSURE

**Figure 7:**
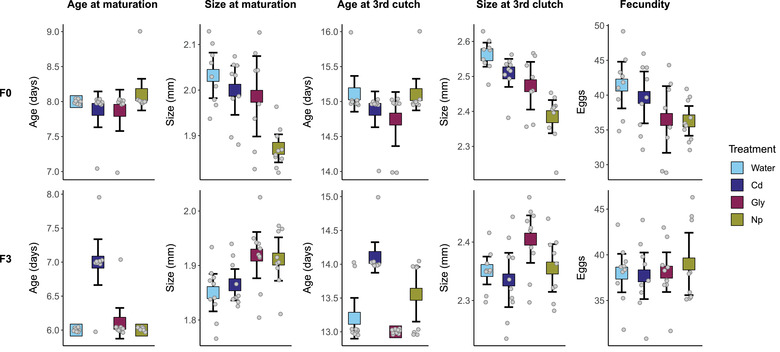
Life history trait values (mean ± 95% confidence interval) for individuals in the polluted generation (F0) and three generations after return to clean water (F3) are shown for water and the three pollutants. Small gray points represent measured values, and have been jittered horizontally for all traits, and vertically for age traits to aid with interpretation.

Chronic exposure to sublethal doses of pollutants led to the disruption of life‐history traits relative to populations in clean water (Fig 7; Table S5). Direct exposure to pollutants (F0 individuals) significantly influenced *D. pulex* size at maturity (*F*_(3, 32)_ = 8.93, FDR = 0.0005) and size at third clutch (*F*_(3, 32)_ = 13.73, FDR < 0.0001), with Np‐treated individuals significant smaller than controls at both life stages (Dunnett's tests; maturity: *P* < 0.0001; third clutch: *P* < 0.0001) and Gly‐treated individuals significantly smaller than controls at the third clutch (Dunnett's test; *P* = 0.0165).

When polluted populations were returned to clean water, significant perturbations to growth and development rates remained observable in great grand offspring (F3). Treatment exerted a significant negative effect on age at maturity (*F*_(3, 35)_ = 28.151, FDR < 0.0001) and age at third clutch (*F*_(3, 35)_ = 17.22, FDR < 0.0001), with Cd‐treated individuals older than controls at both life stages (Dunnett's tests; maturity: *P* < 0.0001; third clutch: *P* < 0.0001). Treatment also influenced size at maturity in the F3 (*F*_(3, 35)_ = 4.5058, FDR = 0.0179), but unexpectedly led to larger sizes. Both Np‐treated (Dunnett's test; *P* = 0.0117) and Gly‐treated individuals (Dunnett's test; *P* = 0.0312) were larger than controls at this life stage.

## Discussion

Consistent with previous results in *Daphnia*, we found that overall methylation of CpGs in the clonal crustacean *Daphnia pulex* was low (Asselman et al. 2015, 2016; Kvist et al. 2018). Nonetheless, CpGs with higher levels of methylation (>50%) tended to be in exons or promotor regions (2 kb upstream of the first exon), suggesting that in *D. pulex* methylation could either play a functional role or be involved in nucleosome positioning and chromatin formation, as suggested by Lewis et al. (2020). Long‐term exposure (>15 generations) to three pollutants induced an epigenetic memory that lasted for many generations (>15) after the pollutant stress ended. Some of these epigenetic modifications were shared by *D. pulex* still experiencing the pollutant stress, suggesting that they were “persistent” and may be stably transmitted over many generations. Other changes to DNA methylation appeared to be a “direct” response to the environmental stress and were absent when the pollutant was no longer experienced. These modifications seem to be reversible, disappearing after successive reproductive events (Burggren 2015). A third class of DM was observed only in those *D. pulex* that no longer experienced the pollutant stress and appear to be a “legacy” of prior pollutant exposure. Differentially methylated CpGs tended to be in genes with comparatively high levels of methylation, and “persistent” modifications occurred in genes with the highest methylation. Interestingly, although “persistent” modifications tended to show reduced methylation compared with controls, “legacy” modifications were more likely to show increases in methylation. There was a large overlap in the genes that were differentially methylated between pollutants, supporting the idea that some changes to global methylation are part of a general stress response (Jeremias et al. 2018). The observation of epigenetic changes being stably transmitted across many reproductive generations provides support for the idea that the environmentally sensitive epigenome plays an important role in phenotypic evolution (Adrian‐Kalchhauser et al. 2020).

### MAINTENANCE OF EPIGENETIC CHANGES OVER MANY GENERATIONS

Until relatively recently, few studies had observed environmentally induced epigenetic modifications persisting in animals beyond the directly affected generations (i.e., the developing embryos or the germline within an embryo; Heard and Martienssen 2014). Now there is increasing evidence that generations with no direct exposure to an environmental stimulus still display signs of transgenerational epigenetic modification (Jeremias et al. 2018; Kamstra et al. 2018; Beck et al. 2021). Yet the observation of epigenetic modifications persisting for so many generations (>15) after an environmental stimulus is rare (for an exception, see Klosin et al. 2017). Modifications may persist longer in parthenogenetic organisms like *Daphnia* due to the lack of meiotic division and fertilization (Jablonka and Raz 2009). Yet although parthenogenesis precludes generational epigenetic changes associated with fertilization (He et al. 2011) and parental imprinting (Head 2014), epigenetic reprogramming still occurs during gametogenesis, embryogenesis, and organogenesis (Vogt 2017; Li et al. 2019; Bicho et al. 2020a), processes that are not dependent on reproductive mode. In parthenogenetic *Daphnia*, histone modifications (Robichaud et al. 2012) and DNA methylation (Kvist et al. 2020) change dynamically during development. Our observation that environmentally induced epigenetic modifications are transmitted across many generations, despite this epigenetic reprogramming, suggests that these transgenerational effects have the potential to influence evolution.

Recent evidence from mammals suggests that epigenetic marks removed during very early development can be reinstated with significant fidelity (Kremsky and Corces 2020). If similar mechanisms operate in invertebrates, this could allow environmentally induced modifications to be stably transmitted between generations. Epigenetic marks are likely to exist on a continuum of stability (Heckwolf et al. 2020), with some highly stable and others “washing‐in” and “washing‐out” over successive generations (Burggren 2015; Eirin‐Lopez and Putnam 2019). What determines this stability is poorly understood; however, our finding that persistent epigenetic modifications showed higher methylation than direct (reversible) ones suggests that factors such as the initial methylation state of the CpG may influence the stability of transgenerational transmission.

Our observation of increased methylation in legacy CpGs also bears further investigation. Although reduced methylation is consistent with previous ecotoxicology results in *Daphnia* (Athanàsio et al. 2018; Jeremias et al. 2018; Trijau et al. 2018), increases in methylation are more unusual. Among vertebrates, global increases in methylation can occur in response to pollutant‐induced DNA damage (Qiu et al. 2019) and can lead to carcinogenesis (Wang et al. 2015). In invertebrates, pollutant‐induced increases in methylation have also been observed (Nica et al. 2017) and may persist in unexposed generations (Bicho et al. 2020b). Increased methylation of some legacy CpGs could therefore be a marker of genetic variation caused by mutation or DNA damage, or if methylation and transcription are linked, it may be part of a compensatory response to the reduced methylation at other sites in the switch treatment (i.e., persistent CpGs). These are speculative hypotheses, and the functional importance of increased methylation remains unclear; however, the fact that we observed such a response for all three pollutants suggests that this may be a general long‐term consequence of exposure to pollutants.

### FUNCTIONAL GENOMIC CONTEXT OF DNA METHYLATION

In line with other results from *Daphnia* and many other arthropods (Lewis et al. 2020), we found low overall levels of DNA methylation, although highly methylated CpG sites were overrepresented in both promotors and exons. The suppressive effect of DNA methylation on promotor transcription is a relatively well‐studied phenomenon in vertebrates (Feng et al. 2010) but significant methylation of promotors is rare in invertebrates (Lewis et al. 2020). Although Asselman et al ( 2017) also found promoters and exons of *D. magna* to be more methylated (and more likely to contain differentially methylated CpGs) than introns, Kvist et al. (2018) did not report promoter methylation in either *D. magna* or *D. pulex*. Their estimate of global methylation in *D. pulex* was also considerably lower (0.41–0.44%) than ours (1.87%). Differences in promoter methylation may have arisen through variation in the regions that were considered promoters (defined as 1, 1.5, and 2 kb upstream of exon 1 in Kvist et al. 2018, Asselman et al. 2017, and this study, respectively), whereas differences in global methylation could reflect both sequencing variation and genotypic variation: Kvist et al. reported considerable variation in global methylation between two genotypes of *D. magna* (1.03% and 1.51%) from different populations sequenced at different times.

Outside of gene bodies, methylation tends to be low in invertebrates, whereas many vertebrate genomes are heavily methylated, with small areas of hypomethylation around promoters (Keller et al. 2016). However, rather than strictly demarcating the evolution of vertebrates, these differences in methylation may represent gradual transitions among deuterostomes (Okamura et al. 2010), perhaps associated with evolutionary innovations during embryogenic reprogramming (Xu et al. 2019), and even among protostomes, genome methylation is highly variable (Lewis et al. 2020). Although patterns of global and promoter methylation vary across different animal taxa, gene body methylation (GbM) of exons appears to be a conserved phenomenon (Zilberman 2017; Männer et al. 2021), albeit one with unclear functional consequences (Lewis et al. 2020). Among arthropods, there is some evidence that GbM can affect gene expression (e.g., Flores et al. 2012; Gatzmann et al. 2018), but GbM does not always correlate with expression (Marshall et al. 2019), and removal of methylation may have little effect on gene expression in adult tissue (Bewick et al. 2019). Increasing evidence suggests that any functional consequences of GbM may occur through interactions with the nucleosome and chromatin (Lou et al. 2014; Lewis et al. 2020), for example, by promoting histone acetylation at transcription start sites (Xu et al. 2021). Although we did not explore the relationship between GbM and gene expression, the GO biological process “gene‐expression” was significantly overrepresented for direct responses in all three pollutants and persistent responses in Cd and Np, suggesting that pollutant‐induced methylation changes may influence transcription. Beyond gene expression, functionally enriched terms were not shared across more than two pollutant/response types. Furthermore, changes in methylation generally resulted in overrepresentation of rather generic GO terms. Although we did not find any enriched nongeneric GO terms (those associated with <2000 associated *D. pulex* uniprot IDs) that were shared among the three persistent responses, this was not due to a lack of overlap in persistently DM *D. pulex* genes. As demonstrated by the set (Venn diagram) analysis, there was a large degree of overlap in many of the persistently DM genes. Rather than sharing a common function, these genes may be linked by having higher baseline levels of methylation in controls, making them more susceptible to demethylation following pollutant exposure. Chemical stress is likely to affect DNA methylation via disruption of DNA methyltransferase activity (Takiguchi et al. 2003; Šrut 2021). It has been suggested that a shortage of the methyl donor *S*‐adenosylmethionine (Lee et al. 2009), which is also required in certain detoxification pathways (Mirbahai and Chipman 2014), may contribute to pollutant‐induced demethylation. A recent study of Cd‐exposure in the crustacean *Gammarus fossarum* by Cribiu et al. (2018) found that initially (after 14 days of exposure) individuals showed reduced methylation, but that subsequently (after 1 month of exposure) global methylation increased to a level greater than controls, potentially caused by an overcompensation in DNA methyltransferase activity (Šrut 2021). Although the experiments of Cribiu et al. (2018) were performed on individuals, if such compensatory effects also operate across generations, it could help to explain why some direct DM and the majority of legacy DM effects lead to increased methylation.

Although functional enrichment rarely involved specific GO terms, an exception was in the case of the Cd legacy response, where the terms “endonucleolytic cleavage of tricistronic rRNA transcript (SSU‐rRNA, 5.8S rRNA, LSU‐rRNA)” and “chemotaxis” were overrepresented. rRNA genes frequently show high levels of methylation in vertebrates (Stancheva et al. 1997) and invertebrates (Falckenhayn et al. 2013), with methylation influencing chromatin and nucleolus structure (McKeown and Shaw 2009) in a developmentally dynamic way (Gupta and Santoro 2020). Chemotaxis plays a critical role in cell migration during development, as does neurogenesis (also overrepresented in the Cd legacy response). We therefore speculate that Cd legacy responses include effects on development. This also matches with one of the phenotypic results, in which F3 *D. pulex* (exposed to Cd three generations earlier) were the only group that showed significant reductions in age at maturity and age at third clutch.

### PHENOTYPIC AND EVOLUTIONARY CONSEQUENCES

As well as observing pollutant‐induced epigenetic modifications in directly exposed generations of *D. pulex*, we also observed significant pollutant‐induced phenotypic changes in unexposed generations. As well as the negative effects on age at maturity and age at third clutch in descendants of Cd‐treated *D. pulex*, there appeared to be positive effects of Gly and Np treatment on size at maturity in descendants, although there was no accompanying increase in fecundity, which often correlates with body size in *Daphnia* when food is nonlimiting (Taylor and Gabriel 1992). Although it is difficult to speculate on the cause of these phenotypic effects, we note that size at maturity was reduced in the directly affected F0, with an apparent compensatory response in the F3. Such compensatory response to toxins has been observed in *C. elegans*, where increased histone methylation was proposed to play a role (Yue et al. 2021). Because the methylation and phenotypic experiments were carried out separately, we cannot directly link the observed long‐lasting epigenetic changes to significant life history effects. However, it seems likely that similar modifications to the epigenome occurred in the phenotypic experiment and may have contributed to the observed phenotypic differences.

Epigenetic inheritance has been predicted to play a role in facilitating transgenerational phenotypic responses (adaptive or otherwise) to environmental change (Jablonka and Lamb 2005; Bonduriansky and Day 2009; Day and Bonduriansky 2011). Evidence is now emerging that varied epigenetic processes including DNA methylation facilitate transgenerational plasticity and influence phenotypic evolution (Adrian‐Kalchhauser et al. 2020; Heckwolf et al. 2020; Hu et al. 2021). Some early models of transgenerational epigenetic inheritance highlighted the transience of epigenetic marks as a key feature facilitating the evolution of short‐term environment‐induced phenotypic changes that can be reversed in the absence of the environment or stressor (Cropley et al. 2012; Burggren 2014). In contrast, other models found that the optimal stability of nongenetically transmitted variants across generations depends on environmental periodicity, with greater stability favored when fluctuations span a greater number of generations (Lachmann and Jablonka 1996). As empirical studies of epigenetic mechanisms increase, there is a realization that nongenetic inheritance mechanisms are often much more complicated than we first imagined, involving numerous diverse pathways and complex interactions between genetic and nongenetic factors (Adrian‐Kalchhauser et al. 2020). The diversity of DNA methylation responses (direct, persistent, and legacy) found in this study supports this idea. Although direct responses appear to be quite transient and therefore of less evolutionary consequence, persistent environmentally induced epigenetic changes lasting for many generations are likely to be important in evolutionary terms. If variation in such stably transmitted epigenetic modifications is associated with phenotypic variation that can be selected upon, it could conceivably fuel a rapid evolutionary response based on environment‐induced meQTL variation (Hu et al. 2021). Such a mechanism might be especially likely to evolve in an asexual organism such as *Daphnia* where new environments are often colonized by a few individuals and genetic bottlenecks and founder effects may be common (Haag et al. 2005). The evolutionary importance of legacy responses is less clear but as well as highlighting their potential role in compensating for persistent effects, we speculate that they could be part of the system that preserves the memory of previous DNA methylation marks across generations (Adrian‐Kalchhauser et al. 2020), or they might be associated with a mechanism for altering the accessibility and mutability of DNA itself (Burggren 2015). Many epigenomic marks are also likely to be determined by DNA sequence variation and differ among individual genotypes (Garg et al. 2018; Martin‐Trujillo et al. 2020). Improving our understanding of genome‐epigenome interactions, as well as the mechanisms by which epigenetic modifications influence development and physiology (Perez and Lehner 2019), remains a key challenge in determining whether such effects may play an adaptive role in rapid evolution. Studies that measure changes in methylation, transcription, and developmental plasticity, as well as incorporating single‐cell epigenomics and transcriptomics, will be well placed to identify the functional consequences of environmentally induced epigenetic change (Navarro‐Martín et al. 2020).

### AUTHOR CONTRIBUTIONS

SJP, DB, and SP obtained funding. SP, HC, DB, and SJP designed the experiments and interpreted the results. SP, HC, and EH performed bioinformatic and statistical analysis. SP, EH, and SJP wrote this article. HC and BC performed laboratory assays.

### DATA ARCHIVING

Genomic data has been deposited at the European Nucleotide Archive (ENA: https://www.ebi.ac.uk/ena) with the project accession PRJEB47706. Phenotypic data and processed genomic data, together with R scripts for their analysis, have been deposited at Dryad (https://doi.org/10.5061/dryad.h9w0vt4k9).

## Supporting information


**Table S1**. Statistical significance of concentrations of cadmium (0.05, 0.1, and 1 μg L‐1), glyphosate (10, and 50 μg L‐1) and 4‐nonylphenol (5, and 25 μg L‐1) on age at maturity, size at, age at first clutch, and fecundity during the first three clutches measured during preliminary studies.
**Table S2**. Statistical results testing whether response type (curtox direct, curtox persistent, switch persistent or switch legacy), genomic feature (exon, intron, 5’−2kb and 3’−2kb) or their interaction significantly influenced A) the difference in methylation % between treatments and controls for DM CpGs or B) the methylation % of DM CpGs in untreated controls.
**Table S3**. Estimated marginal means (emmeans) for significant factors in the linear model testing for effects of response type (curtox direct, curtox persistent, switch persistent or switch legacy) and genomic feature (exon, intron, 5’−2kb and 3’−2kb) on the difference in methylation % between treatments and controls for DM CpGs.
**Table S4**. Pairwise contrasts for significant factors in the glm testing for effects of response type (curtox direct, curtox persistent, switch persistent or switch legacy) and genomic feature (exon, intron, 5’−2kb and 3’−2kb) on the methylation % of DM CpGs in untreated controls.
**Table S5**. Results of ANOVAs testing for effects of pollutant treatment on 5 phenotypic traits in the directly exposed generation (F0) and the great‐grand‐offspring generation (F3), which did not directly experience cues associated with pollutant.
**Table S6**. Dunnett's Multiple Comparison post‐hoc test results for traits that were found to be significantly affected by pollutant treatment compared to controls (see Table S4).
**Figure S1**. Effects of different concentrations of cadmium (A, D G, J), glyphosate (B, E, H, K) and 4‐nonylphenol (C, F, I, L) on age at maturity (A‐C), size at maturity (D‐F), age at first clutch (G‐I), and fecundity during the first three clutches (J‐L).
**Figure S2**. CpG density surrounding CpG sites with either greater than or less than 50% methylation in controls.
**Figure S3**. Results of repeatability analysis using 6 control samples.
**Figure S4**. Distribution of significantly differentially methylated CpGs in A) Cd curtox, B) Cd switch, C) Gly curtox, D) Gly switch, E) Np curtox and F) Np switch treatments.
**Figure S5**. Overrepresentation of gene ontology (GO) biological process terms in DM genes that showed decreased (−) or increased (+) methylation for the two treatments (curtox and switch). DM genes from the three different pollutants were combined.
