# Supplementary material for: Pollution induces epigenetic effects that are stably transmitted across multiple generations
Source: Evol Lett. 2022 Feb 3;6(2):118–35. doi: 10.1002/evl3.273 (PMC8966472; doi:10.1002/evl3.273)
Supplement: Supplementary file 1 — Table S1. Statistical significance of concentrations of cadmium (0.05, 0.1, and 1 μg L‐1), glyphosate (10, and 50 μg L‐1) and 4‐nonylphenol (5, and 25 μg L‐1) on age at maturity, size at, age at first clutch, and fecundity during the first three clutches measured during preliminary studies. Table S2. Statistical results testing whether response type (curtox direct, curtox persistent, switch persistent or switch legacy), genomic feature (exon, intron, 5’−2kb and 3’−2kb) or their interaction significantly influenced A) the difference in methylation % between treatments and controls for DM CpGs or B) the methylation % of DM CpGs in untreated controls. Table S3. Estimated marginal means (emmeans) for significant factors in the linear model testing for effects of response type (curtox direct, curtox persistent, switch persistent or switch legacy) and genomic feature (exon, intron, 5’−2kb and 3’−2kb) on the difference in methylation % between treatments and controls for DM CpGs. Table S4. Pairwise contrasts for significant factors in the glm testing for effects of response type (curtox direct, curtox persistent, switch persistent or switch legacy) and genomic feature (exon, intron, 5’−2kb and 3’−2kb) on the methylation % of DM CpGs in untreated controls. Table S5. Results of ANOVAs testing for effects of pollutant treatment on 5 phenotypic traits in the directly exposed generation (F0) and the great‐grand‐offspring generation (F3), which did not directly experience cues associated with pollutant. Table S6. Dunnett's Multiple Comparison post‐hoc test results for traits that were found to be significantly affected by pollutant treatment compared to controls (see Table S4). Figure S1. Effects of different concentrations of cadmium (A, D G, J), glyphosate (B, E, H, K) and 4‐nonylphenol (C, F, I, L) on age at maturity (A‐C), size at maturity (D‐F), age at first clutch (G‐I), and fecundity during the first three clutches (J‐L). Figure S2. CpG density surrounding CpG sites with [file EVL3-6-118-s001.docx]

Supplementary Text and Figures

**Pollution induces epigenetic effects that are stably transmitted across multiple generations**

Ewan Harney ^†^, Steve Paterson ^†^, Hélène Collin, Brian H.K. Chan, Daimark Bennett and Stewart J. Plaistow

† These authors contributed equally.

**Table S1.** Statistical significance of concentrations of cadmium (0.05, 0.1, and 1 μg L-1), glyphosate (10, and 50 μg L-1) and 4-nonylphenol (5, and 25 μg L-1) on age at maturity, size at, age at first clutch, and fecundity during the first three clutches measured during preliminary studies. D. pulex were maintained individually during these experiments. Assays were carried out for three different D. pulex clones (LL14, LL18 and LL23 for Cd, and LL14, LL18 and LL28 for Gly and Np). Linear mixed effects models (considering pollutant as a fixed factor and clone as a random factor) were fit to the data (for fecundity, a generalized linear mixed effects model with a Poisson distribution was used) in R using the lme4 package. The significance of pollutant was determined through a likelihood ratio test of models with and without pollutant included. When pollutant was found to be significant (P < 0.05), post-hoc tests were performed comparing different levels of the pollutants to controls. Post-hoc tests for lmers and glmers were carried out in the emmeans package to control for multiple comparisons. Significance is indicated by the following symbols: · P < 0.1; * P < 0.05; ** P < 0.001; *** P < 0.0001.

| Trait | Pollutant | Significance of pollutant in model | | | | | |  | Post-hoc tests of pollutant concentration | | | | | | |
| --- | --- | --- | --- | --- | --- | --- | --- | --- | --- | --- | --- | --- | --- | --- | --- |
|  |  | df | AIC (treat) | AIC (null) | LRT | P (Chi) | Sig. |  | Contrast (vs ctrl) | Estimate | SE | df | T ratio | P value | Sig. |
| age at maturity | cd | 3 | 84.89 | 79.31 | 0.42 | 0.9354 |  |  |  |  |  |  |  |  |  |
|  | gly | 2 | 94.65 | 92.39 | 1.74 | 0.4182 |  |  |  |  |  |  |  |  |  |
|  | np | 2 | 96.96 | 98.30 | 5.34 | 0.0694 | · |  |  |  |  |  |  |  |  |
| size at maturity | cd | 3 | -119.94 | -120.11 | 5.83 | 0.1201 |  |  |  |  |  |  |  |  |  |
|  | gly | 2 | -136.51 | -135.13 | 5.38 | 0.0678 | · |  |  |  |  |  |  |  |  |
|  | np | 2 | -168.71 | -144.34 | 28.37 | < 0.0001 | *** |  | 5 | -0.031 | 0.022 | 79.1 | -1.39 | 0.2888 |  |
|  |  |  |  |  |  |  |  |  | 25 | -0.122 | 0.022 | 79.1 | -5.46 | < 0.0001 | *** |
| age at first clutch | cd | 3 | 71.37 | 82.14 | 16.78 | 0.0008 | *** |  | 0.05 | -0.513 | 0.147 | 48.1 | -3.50 | 0.0029 | ** |
|  |  |  |  |  |  |  |  |  | 0.1 | -0.528 | 0.150 | 48.1 | -3.53 | 0.0027 | ** |
|  |  |  |  |  |  |  |  |  | 1 | -0.491 | 0.154 | 48.1 | -3.19 | 0.0072 | ** |
|  | gly | 2 | 116.49 | 120.52 | 8.03 | 0.0180 | * |  | 10 | 0.087 | 0.119 | 77 | 0.73 | 0.6816 |  |
|  |  |  |  |  |  |  |  |  | 50 | -0.243 | 0.122 | 77 | -2.00 | 0.0921 | · |
|  | np | 2 | 108.29 | 116.10 | 11.81 | 0.0027 | ** |  | 5 | 0.020 | 0.111 | 79 | 0.18 | 0.9679 |  |
|  |  |  |  |  |  |  |  |  | 25 | 0.342 | 0.111 | 79 | 3.09 | 0.0055 | · |
| total fecundity | cd | 3 | 333.87 | 335.36 | 7.50 | 0.0576 | · |  |  |  |  |  |  |  |  |
|  | gly | 2 | 562.67 | 580.31 | 21.64 | < 0.0001 | *** |  | 10 | -0.184 | 0.041 | - | -4.56 | < 0.0001 | *** |
|  |  |  |  |  |  |  |  |  | 50 | -0.126 | 0.041 | - | -3.11 | 0.0037 | ** |
|  | np | 2 | 553.92 | 575.34 | 25.42 | < 0.0001 | *** |  | 5 | -0.031 | 0.039 | - | -0.78 | 0.644 |  |
|  |  |  |  |  |  |  |  |  | 25 | -0.191 | 0.041 | - | -4.66 | < 0.0001 | *** |

**Table S2.** Statistical results testing whether response type (curtox direct, curtox persistent, switch persistent or switch legacy), genomic feature (exon, intron, 5’-2kb and 3’-2kb) or their interaction significantly influenced A) the difference in methylation % between treatments and controls for DM CpGs or B) the methylation % of DM CpGs in untreated controls. For A) linear models were fitted to each pollutant, while for B) generalized linear models with a quasibinomial distribution were fitted to each pollutant. Differences between the levels of response and feature are shown in supplementary tables 2 and 3.

| **Dependent variable** | **Pollutant** | **Term** | **Df** | **Deviance** | **Residual Df** | **Residual deviance** | **F value** | **P value** | **Significance** |
| --- | --- | --- | --- | --- | --- | --- | --- | --- | --- |
| A) Difference in methylation between treatment and control | Cadmium | Null | 1272 | 116.95 |  |  |  |  |  |
|  |  | Response | 3 | 11.6381 | 1269 | 105.32 | 46.6936 | <0.0001 | *** |
|  |  | Feature | 3 | 0.4367 | 1266 | 104.88 | 1.7521 | 0.1545 |  |
|  |  | Response:Feature | 9 | 0.4467 | 1257 | 104.43 | 0.5974 | 0.8 |  |
|  | Gyphosate | Null | 1088 | 73.423 |  |  |  |  |  |
|  |  | Response | 3 | 4.8088 | 1085 | 68.614 | 25.1938 | <0.0001 | *** |
|  |  | Feature | 3 | 0.1353 | 1082 | 68.479 | 0.7089 | 0.5468 |  |
|  |  | Response:Feature | 9 | 0.2098 | 1073 | 68.269 | 0.3664 | 0.9511 |  |
|  | 4-nonylphenol | Null | 948 | 78.511 |  |  |  |  |  |
|  |  | Response | 3 | 10.1975 | 945 | 68.314 | 47.7221 | <0.0001 | *** |
|  |  | Feature | 3 | 0.7579 | 942 | 67.556 | 3.547 | 0.01419 | * |
|  |  | Response:Feature | 9 | 1.0999 | 933 | 66.456 | 1.7157 | 0.0812 | · |
| B) Methylation of controls | Cadmium | Null | 1272 | 583.92 |  |  |  |  |  |
|  |  | Response | 3 | 31.0672 | 1269 | 552.85 | 25.9066 | <0.0001 | *** |
|  |  | Feature | 3 | 4.3384 | 1266 | 548.51 | 3.6177 | 0.01279 | * |
|  |  | Response:Feature | 9 | 1.9294 | 1257 | 546.58 | 0.5363 | 0.84879 |  |
|  | Gyphosate | Null | 1088 | 546.91 |  |  |  |  |  |
|  |  | Response | 3 | 36.676 | 1085 | 510.23 | 27.3062 | <0.0001 | *** |
|  |  | Feature | 3 | 3.416 | 1082 | 506.82 | 2.543 | 0.0549 | · |
|  |  | Response:Feature | 9 | 2.624 | 1073 | 504.19 | 0.6512 | 0.7535 |  |
|  | 4-nonylphenol | Null | 948 | 477.67 |  |  |  |  |  |
|  |  | Response | 3 | 56.521 | 945 | 421.15 | 45.5113 | <0.0001 | *** |
|  |  | Feature | 3 | 2.457 | 942 | 418.69 | 1.9781 | 0.11564 |  |
|  |  | Response:Feature | 9 | 6.901 | 933 | 411.79 | 1.8521 | 0.05564 | · |

**Table S3.** Estimated marginal means (emmeans) for significant factors in the linear model testing for effects of response type (curtox direct, curtox persistent, switch persistent or switch legacy) and genomic feature (exon, intron, 5’-2kb and 3’-2kb) on the difference in methylation % between treatments and controls for DM CpGs. Significant negative emmean values (e.g., cadmium curt_pers) suggest that methylation of DM CpGs tended to decrease, significant positive values (e.g., cadmium swit-lega) suggest that methylation DM CpGs tended to increase.

| **Pollutant** | **Term** | **Term level** | **emmean** | **SE** | **df** | **z ratio** | **P value** | **Sig.** |
| --- | --- | --- | --- | --- | --- | --- | --- | --- |
| Cadmium | Response | curt_dire | -0.00954 | 0.0155 | Inf | -0.615 | 0.5384 |  |
|  |  | curt_pers | -0.13659 | 0.0173 | Inf | -7.891 | <0.0001 | *** |
|  |  | swit_pers | -0.13122 | 0.0174 | Inf | -7.526 | <0.0001 | *** |
|  |  | swit_lega | 0.09132 | 0.0148 | Inf | 6.163 | <0.0001 | *** |
| Glyphosate | Response | curt_dire | 0.0859 | 0.0181 | Inf | 4.744 | <0.0001 | *** |
|  |  | curt_pers | -0.0755 | 0.016 | Inf | -4.721 | <0.0001 | *** |
|  |  | swit_pers | -0.0706 | 0.0156 | Inf | -4.535 | <0.0001 | *** |
|  |  | swit_lega | 0.0422 | 0.0128 | Inf | 3.306 | 0.0009 | *** |
| 4-nonylphenol | Response | curt_dire | 0.0291 | 0.016 | Inf | 1.824 | 0.0681 |  |
|  |  | curt_pers | -0.1574 | 0.0169 | Inf | -9.314 | <0.0001 | *** |
|  |  | swit_pers | -0.1628 | 0.0176 | Inf | -9.262 | <0.0001 | *** |
|  |  | swit_lega | 0.0713 | 0.0202 | Inf | 3.538 | 0.0004 | *** |
|  | Feature | 5'-2kb | -0.0236 | 0.017 | Inf | -1.389 | 0.1647 |  |
|  |  | exon | -0.067 | 0.014 | Inf | -4.774 | <0.0001 | *** |
|  |  | intron | -0.1106 | 0.0239 | Inf | -4.619 | <0.0001 | *** |
|  |  | 3'-2kb | -0.037 | 0.019 | Inf | -1.948 | 0.0514 |  |

**Table S4.** Pairwise contrasts for significant factors in the glm testing for effects of response type (curtox direct, curtox persistent, switch persistent or switch legacy) and genomic feature (exon, intron, 5’-2kb and 3’-2kb) on the methylation % of DM CpGs in untreated controls. Significant negative estimates (e.g. cadmium curt-dire - curt_pers) suggest that among control *D. pulex*, DM CpGs classed as curtox direct had lower methylation than DM CpGs classed as curtox persistent.

| **Pollutant** | **Term** | **Pairwise contrast** | **estimate** | **SE** | **z ratio** | **P value** | **Sig.** |
| --- | --- | --- | --- | --- | --- | --- | --- |
| Cadmium | Response | curt_dire - curt_pers | -0.5618 | 0.111 | -5.065 | <.0001 | *** |
|  |  | curt_dire - swit_pers | -0.5404 | 0.111 | -4.849 | <.0001 | *** |
|  |  | curt_dire - swit_lega | 0.2357 | 0.112 | 2.11 | 0.1917 |  |
|  |  | curt_pers - swit_pers | 0.0215 | 0.112 | 0.192 | 1 |  |
|  |  | curt_pers - swit_lega | 0.7975 | 0.112 | 7.121 | <.0001 | *** |
|  |  | swit_pers - swit_lega | 0.776 | 0.113 | 6.897 | <.0001 | *** |
|  | Feature | (5' - 2kb) - exon | -0.3093 | 0.102 | -3.018 | 0.0152 | * |
|  |  | (5' - 2kb) - intron | -0.1737 | 0.133 | -1.303 | 0.7232 |  |
|  |  | (5' - 2kb) - (3' - 2kb) | -0.0665 | 0.117 | -0.57 | 0.9936 |  |
|  |  | exon - intron | 0.1356 | 0.123 | 1.107 | 0.8466 |  |
|  |  | exon - (3' - 2kb) | 0.2428 | 0.104 | 2.329 | 0.1135 |  |
|  |  | intron - (3' - 2kb) | 0.1072 | 0.135 | 0.796 | 0.9641 |  |
| Glyphosate | Response | curt_dire - curt_pers | -0.9632 | 0.156 | -6.155 | <.0001 | *** |
|  |  | curt_dire - swit_pers | -0.9744 | 0.155 | -6.286 | <.0001 | *** |
|  |  | curt_dire - swit_lega | -0.1958 | 0.154 | -1.27 | 0.7457 |  |
|  |  | curt_pers - swit_pers | -0.0112 | 0.124 | -0.09 | 1 |  |
|  |  | curt_pers - swit_lega | 0.7674 | 0.123 | 6.219 | <.0001 | *** |
|  |  | swit_pers - swit_lega | 0.7786 | 0.122 | 6.406 | <.0001 | *** |
| 4-nonylphenol | Response | curt_dire - curt_pers | -1.03057 | 0.126 | -8.172 | <.0001 | *** |
|  |  | curt_dire - swit_pers | -1.02489 | 0.128 | -7.988 | <.0001 | *** |
|  |  | curt_dire - swit_lega | 0.15833 | 0.159 | 0.999 | 0.8993 |  |
|  |  | curt_pers - swit_pers | 0.00568 | 0.119 | 0.048 | 1 |  |
|  |  | curt_pers - swit_lega | 1.1889 | 0.151 | 7.87 | <.0001 | *** |
|  |  | swit_pers - swit_lega | 1.18322 | 0.153 | 7.738 | <.0001 | *** |

**Table S5.** Results of ANOVAs testing for effects of pollutant treatment on 5 phenotypic traits in the directly exposed generation (F0) and the great-grand-offspring generation (F3), which did not directly experience cues associated with pollutant.

| **Generation** | **Trait** | **Factor** | **Df** | **Sum Sq.** | **Mean Sq.** | **F-Value** | **P-value** | **FDR** | **Sig.** |
| --- | --- | --- | --- | --- | --- | --- | --- | --- | --- |
| F0 | Age at maturity | Pollutant | 3 | 0.3083 | 0.1028 | 1.23 | 0.3133 | 0.3481 |  |
|  |  | Residuals | 32 | 2.6639 | 0.0832 |  |  |  |  |
|  | Size at maturity | Pollutant | 3 | 0.1407 | 0.0469 | 8.93 | 0.0002 | 0.0005 | *** |
|  |  | Residuals | 32 | 0.1681 | 0.0053 |  |  |  |  |
|  | Age at 3rd clutch | Pollutant | 3 | 0.7944 | 0.2648 | 2.03 | 0.1296 | 0.1620 |  |
|  |  | Residuals | 32 | 4.1778 | 0.1306 |  |  |  |  |
|  | Size at 3rd clutch | Pollutant | 3 | 0.1596 | 0.0532 | 13.73 | 0.0000 | < 0.0001 | *** |
|  |  | Residuals | 32 | 0.1240 | 0.0039 |  |  |  |  |
|  | Fecundity | Pollutant | 3 | 170.65 | 56.883 | 2.80 | 0.0558 | 0.0797 |  |
|  |  | Residuals | 32 | 650.32 | 20.323 |  |  |  |  |
| F3 | Age at maturity | Pollutant | 3 | 6.9974 | 2.3325 | 28.15 | 0.0000 | < 0.0001 | *** |
|  |  | Residuals | 35 | 2.9000 | 0.0829 |  |  |  |  |
|  | Size at maturity | Pollutant | 3 | 0.0343 | 0.0114 | 4.51 | 0.0089 | 0.0179 | * |
|  |  | Residuals | 35 | 0.0887 | 0.0025 |  |  |  |  |
|  | Age at 3rd clutch | Pollutant | 3 | 6.9701 | 2.3234 | 17.22 | 0.0000 | < 0.0001 | *** |
|  |  | Residuals | 35 | 4.7222 | 0.1349 |  |  |  |  |
|  | Size at 3rd clutch | Pollutant | 3 | 0.0272 | 0.0091 | 3.19 | 0.0355 | 0.0592 | · |
|  |  | Residuals | 35 | 0.0997 | 0.0028 |  |  |  |  |
|  | Fecundity | Pollutant | 3 | 8.74 | 2.915 | 0.24 | 0.8709 | 0.8709 |  |
|  |  | Residuals | 35 | 433.00 | 12.371 |  |  |  |  |

**Table S6.** Dunnett’s Multiple Comparison post-hoc test results for traits that were found to be significantly affected by pollutant treatment compared to controls (see Suppl. Table S4).

| **Generation** | **Trait** | **Pollutant** | **diff** | **Lower CI** | **Upper CI** | **P-value** | **Sig.** |
| --- | --- | --- | --- | --- | --- | --- | --- |
| F0 | Size at maturity | CD | -0.03 | -0.1168 | 0.0517 | 0.6620 |  |
|  |  | GLY | -0.05 | -0.1327 | 0.0410 | 0.4320 |  |
|  |  | NP | -0.16 | -0.2422 | -0.0780 | 0.0001 | *** |
|  | Size at 3rd clutch | CD | -0.05 | -0.1245 | 0.0202 | 0.2022 |  |
|  |  | GLY | -0.09 | -0.1633 | -0.0141 | 0.0165 | * |
|  |  | NP | -0.18 | -0.2477 | -0.1067 | < 0.0001 | *** |
| F3 | Age at maturity | CD | 1.00 | 0.6837 | 1.3163 | < 0.0001 | *** |
|  |  | GLY | 0.10 | -0.2163 | 0.4163 | 0.7801 |  |
|  |  | NP | 0.00 | -0.3250 | 0.3250 | 1.0000 |  |
|  | Size at maturity | CD | 0.01 | -0.0409 | 0.0697 | 0.8603 |  |
|  |  | GLY | 0.07 | 0.0135 | 0.1241 | 0.0117 | * |
|  |  | NP | 0.06 | 0.0047 | 0.1184 | 0.0312 | * |
|  | Age at 3rd clutch | CD | 0.90 | 0.4964 | 1.3036 | < 0.0001 | *** |
|  |  | GLY | -0.20 | -0.6036 | 0.2036 | 0.4855 |  |
|  |  | NP | 0.36 | -0.0591 | 0.7703 | 0.1064 |  |


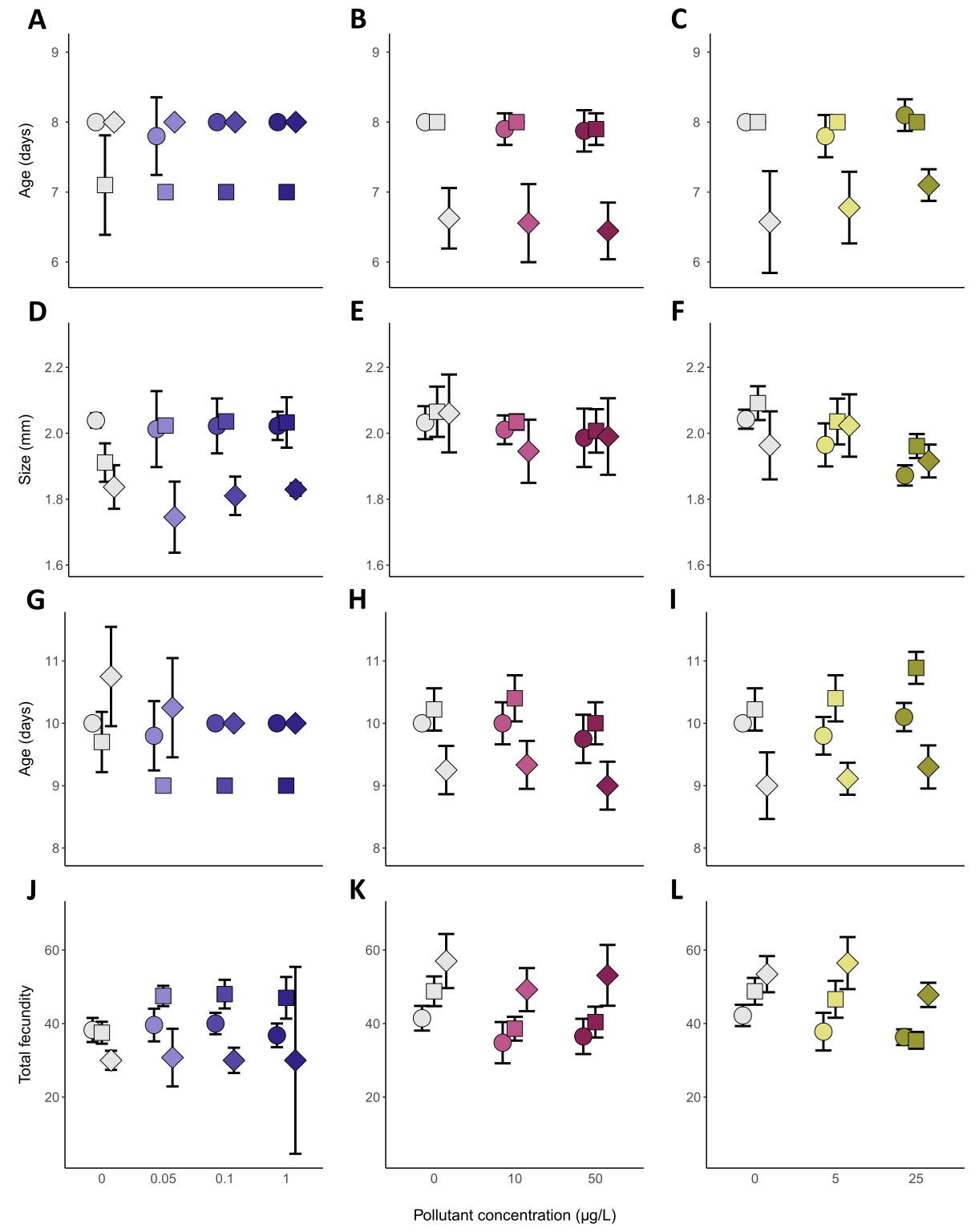


**Figure S1.** Effects of different concentrations of cadmium (A, D G, J), glyphosate (B, E, H, K) and 4-nonylphenol (C, F, I, L) on age at maturity (A-C), size at maturity (D-F), age at first clutch (G-I), and fecundity during the first three clutches (J-L). These preliminary assays were carried out for individually kept *D. pulex* of three different clonal genotypes: LL14, LL18 and LL23 for Cd, and LL14, LL18 and LL28 for Gly and Np. Mean clonal trait values are represented by circles, squares and diamonds respectively, with 95% confidence interval error bars. These assays were not performed simultaneously, so controls (concentration = 0 μg L^-1^) were repeated separately for each pollutant.

**Figure S2.** CpG density surrounding CpG sites with either greater than or less than 50% methylation in controls. Density was calculated from the frequency of CpG sites within 1Kbp on either side of each CpG site within the genome.


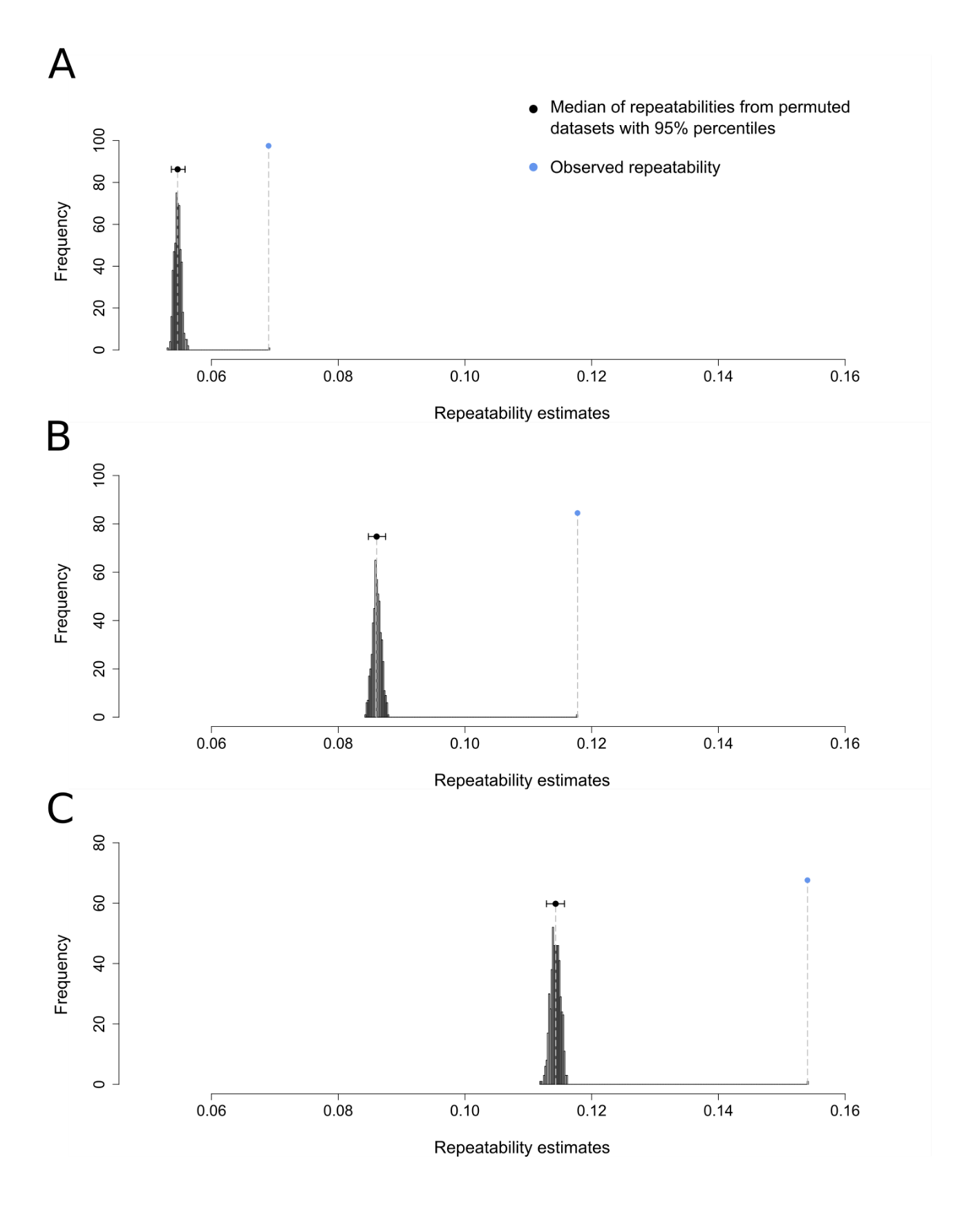


**Figure S3.** Results of repeatability analysis using 6 control samples. Estimates of repeatability for methylation in a random sample of 10,000 CpGs with A) 1-99% variability; B) 5-95% variability and C) 10-90% variability were compared to null distribution (repeatabilities from 500 permuted datasets). In all cases measures of CpG methylation were found to be more repeatable than the permuted distributions, although excluding low variability CpGs resulted in higher repeatability estimates.


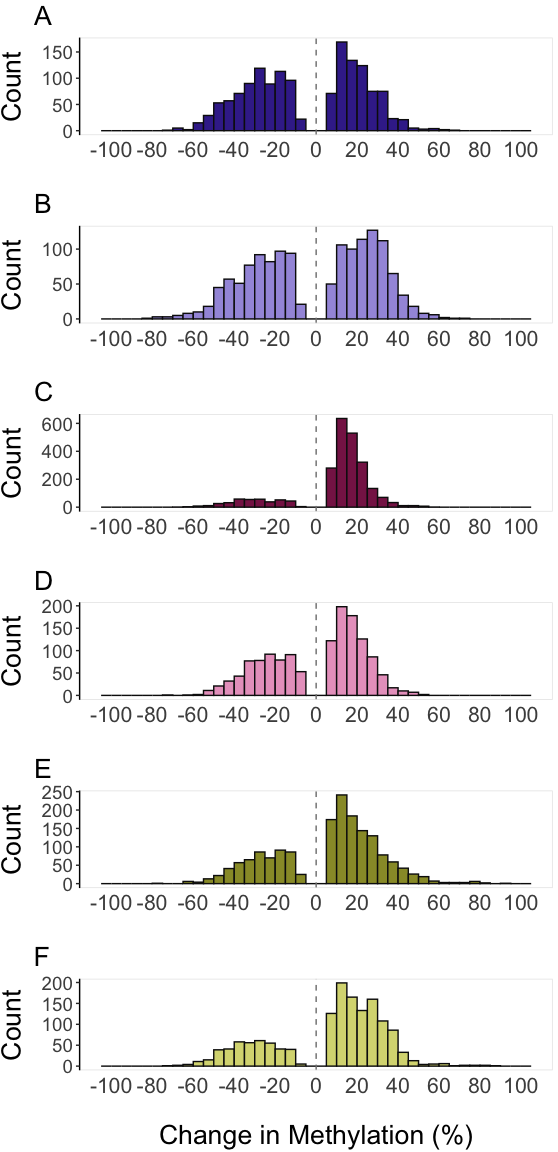


**Figure S4.** Distribution of significantly differentially methylated CpGs in A) Cd curtox, B) Cd switch, C) Gly curtox, D) Gly switch, E) Np curtox and F) Np switch treatments. The change in methylation is the percentage of methylated reads in the treatment reads minus the percentage of methylated reads in the control, as calculated for all DM CpGs. The y-axis scales differ between subplots, allowing a better view of the distribution in each pollutant and treatment combination.


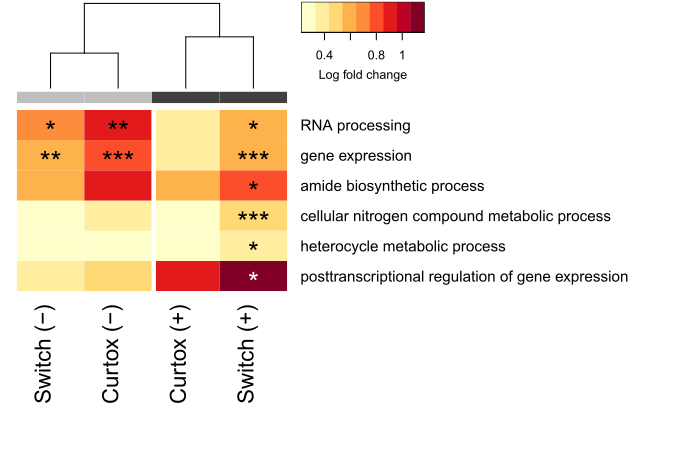


**Figure S5.** Overrepresentation of gene ontology (GO) biological process terms in DM genes that showed decreased (−) or increased (+) methylation for the two treatments (curtox and switch). DM genes from the three different pollutants were combined. Highly generic GO terms associated with ≥ 2000 *D. pulex* genes were removed. Underrepresented terms (fold change in term enrichment < 1, none of which were significant) were set to 1 to better visualise changes in significantly overrepresented terms. Colour intensity denotes log-fold change in term enrichment, which was used to cluster terms and methylation type/treatment classes (also indicated by the greyscale bar underneath the dendrogram). Significance level is indicated by

FDR < 0.001; *** FDR < 0.0001.
